# Editorial: Insights into Microbe–Microbe Interactions in Human Microbial Ecosystems: Strategies to Be Competitive

**DOI:** 10.3389/fmicb.2016.01508

**Published:** 2016-09-23

**Authors:** Nuria Salazar, Clara G. de los Reyes-Gavilán

**Affiliations:** Department of Biochemistry and Microbiology of Dairy Products, Instituto de Productos Lácteos de Asturias, Consejo Superior de Investigaciones CientíficasVillaviciosa, Asturias, Spain

**Keywords:** human microbiota, quorum sensing, gnotobiotic mice, biofilm, *Bifidobacterium*, bacterial-pathogen infection, short chain fatty acids, breast milk

The human body is colonized by trillions of commensal microorganisms (bacteria, archaea, viruses, and microscopic eukaryotes) that are collectively referred to as the human microbiota. The microbiota colonizes the skin and mucosal body surfaces of humans and animals, where they are engaged in a constant crosstalk with the host immune system and metabolism. This human microbiota displays a vast genetic catalog, the so called microbiome, contributing functions that are not encoded by our own human genome (Li et al., 2014). The classical tools to analyze its taxonomy and diversity, such as microscopy and cultivation, have been gradually replaced by culture-independent approaches. Initially the study of the human microbiota focused on taxonomy but interests have shifted to understanding the functional role of these human microbial ecosystems and their implications for the host (Salazar et al., 2014). It is also well established that the composition and functionality of this microbiome is essential for maintaining a “healthy status.” The microbes living on and within the human body inhabit competitive and complex environments, and deploy different ecological strategies for survival, which may imply notable changes on these microorganisms at metabolic, physiological and genetic level.

The current Research Topic covers a collection of reviews, mini-reviews and original research articles that discuss how bacteria adapt to the specific human niches by competing, or otherwise co-existing with other bacteria and host cells. Recent development of high-throughput analytical tools and “meta-omics” technologies has allowed us to obtain complete overviews of community composition and diversity as well as inferred functionality of genes and metabolic pathways in a wide range of body habitats.

The mini-review by Palau-Rodríguez et al. discusses the use of the metabolomic approach as a powerful tool for exploring the crosstalk between microbial and host metabolism in order to identify human gut microbial-host co-metabolites in the context of metabolic diseases such as obesity and type 2 diabetes.

The laboratory mice are useful experimental models for the study of microbial communities in a mammalian host. The review by Martín et al. provides a comprehensive overview of the application of gnotobiotic animals as tools to decipher the mechanisms underlying microbe–microbe and microbe–host interactions. In addition, the combination of gnotobiotic techniques with new approaches (“omics” and genetic engineering) has revealed causative associations between alterations in the commensal microbiota and several diseases.

Cell-cell communication in Firmicutes populations by quorum sensing is mostly mediated by peptides that are released to the extracellular environment. The molecular mechanisms underlying bacterial cell-cell communication are not completely understood in spite of their importance for elucidating the microbial contribution to human health and disease. The mini review of Pérez-Pascual et al. presents the current state of research on the biological relevance in Gram positive bacteria of RRNPP, a family of cytoplasmic transcriptional peptide-associated regulators that modulate the expression of target genes involved to host-microbe interactions and with key roles in the context of commensalism or pathogenesis of certain bacteria in human microbial ecosystems.

It is widely recognized that many bacteria form structured multicellular communities, also known as biofilms. Cell-to-cell interactions lead to the establishment of complex and highly structured communities that are responsible for 75% of human microbial infections. The mini-review of Miquel et al. summarizes strategies for prevention of biofilm growth and biofilm control and focuses on catheter-related infections. The review of García et al. goes deeper into the interaction of pathogenic bacteria with host cells and describes the proteoglycans (PG) family which are complex and ubiquitous host molecules which have a different distribution and composition depending on the tissue, and act as key mediators of bacterial infections. The characterization of PG-pathogen interactions can lead to more effective control of infections, and help to overcome antimicrobial resistance, a world health issue of increasing importance.

The original research of Kaur et al. describes how the bacterium *Pseudomonas aeruginosa* inhibits the growth of *Scedosporium aurantiacum*, an opportunistic fungal pathogen in cystic fibrosis, using a combination of solid plate assays and liquid cultures. The results of this study highlight the importance of biofilm formation by *P. aeruginosa* for inhibiting the growth of *S. aurantiacum* in a mimicked lung environment.

*Clostridium difficile* is an opportunistic pathogen inhabiting the human gut, and is the most frequent aetiological agent of nosocomial diarrhea. Valdés-Varela et al. explore the anti-toxin activity of some *Lactobacillus* and *Bifidobacterium* strains upon the human intestinal epithelial cell line HT29. These two genera are common habitants of the human gastrointestinal tract and some of their members are considered as probiotics.

The human gut microbiome also participates in the biosynthesis and transformation of compounds that are important for both microbial and host physiology. *Bacteroides* are able to use dietary or host-derived glycans as energy sources. In the original research of Rios-Covián et al., the authors have studied the metabolism of the species *Bacteroides fragilis* in the presence of different carbohydrates, including exopolysaccharides synthetized by bifidobacteria. The results show the versatility of *B. fragilis* for adapting to complex carbohydrates and amino acids present in the intestinal environment.

The mini-review by Rios-Covián et al. summarizes the current knowledge on the intestinal microbiota metabolic pathways leading to the production of short chain fatty acids from undigested complex dietary substrates in the gut. Bacterial cross-feeding interactions are involved in the production of a substantial part of these bacterial metabolites, with a huge impact on human health.

The establishment of the gut microbiota is a crucial process influenced by perinatal factors, including the type of infant feeding. Breast milk is considered the optimum food for newborns and health benefits associated with breast-feeding have been reported (Le Huerou-Luron et al., 2010). The research article of Boix-Amorós et al. presents a combined approach to identify the relationships between milk microbiota composition, bacterial load, macronutrients and human cells during lactation using molecular techniques and flow cytometry.

In summary, together the articles of this research topic make a substantial contribution toward understanding the complex interaction among microorganisms residing in human microbial habitats.

## Author contributions

All authors listed, have made substantial, direct and intellectual contribution to the work, and approved it for publication. The editorial was written jointly by the editors of the topic.

## Funding

The research work carried out by Editors of this research topic is being currently funded by project AGL2013-43770-R from Plan Nacional/Plan Estatal de I+D+i (Spanish Ministry of Economy and Competitiveness, MINECO) and by Grant GRUPIN14-043 from “Plan Regional de Investigación del Principado de Asturias.” Both, national and regional grants received cofounding from European Union FEDER funds. NS benefits from a Clarín postdoctoral contract (Marie Slodowska Curie European CoFund Program) cofinanced by Plan Regional de Investigación del Principado de Asturias, Spain.

### Conflict of interest statement

The authors declare that the research was conducted in the absence of any commercial or financial relationships that could be construed as a potential conflict of interest.
